# Recurrence rate of odontogenic keratocyst treated by enucleation and peripheral ostectomy: Retrospective case series with up to 12 years of follow-up

**DOI:** 10.4317/medoral.22366

**Published:** 2018-06-21

**Authors:** Çiğdem Karaca, Kadriye-Ayça Dere, Nuray Er, Alper Aktaş, Emre Tosun, Osman-Taha Köseoğlu, Alp Usubütün

**Affiliations:** 1 DDS, PhD, Hacettepe University, Faculty of Dentistry, Department of Oral and Maxillofacial Surgery, Ankara, Turkey; 2 DDS, 75th Year Oral and Dental Health Hospital, Ankara, Turkey; 3 MD, PhD, Hacettepe University, Faculty of Medicine, Department of Pathology, Ankara, Turkey

## Abstract

**Background:**

Odontogenic keratocysts have been reported with high recurrence rates in the literature so various treatment modalities from simple enucleation to resection have been performed to achieve the cure. The purpose of this retrospective study was to investigate the recurrence rate of odontogenic keratocysts (OKCs) treated by enucleation and peripheral ostectomy.

**Material and Methods:**

An electronic search of the database of the Hacettepe University, Faculty of Medicine, Department of Pathology, was undertaken to identify patients histologically diagnosed with OKCs treated at Department of Oral and Maxillofacial Surgery between 2001 and 2015.

**Results:**

In total, 81 patients were studied. The mean age at the time of diagnosis was 42 years, and the male:female ratio was 1:0.7. OKCs were located primarily in the posterior mandibular region (41%). Twenty-seven patients were re-examined to determine the recurrence rate. The mean follow-up period was 5 years (range, 1–12 years). The recurrence rate was 14.8%. The relationship between location of the lesion and recurrence was not statistically significant (*p* = 0.559). There was also no statistically significant relation between the recurrence rate and treatment option of teeth involved in the lesion (*p* = 0.579).

**Conclusions:**

The authors conclude that treatment of OKCs by enucleation with peripheral ostectomy is associated with minimal morbidity and is preferred over other aggressive treatment modalities. Meticulous radiographic examination and careful surgical resection may decrease the recurrence rate of OKCs.

** Key words:**Odontogenic keratocyst, recurrence rate, enucleation, enucleation plus peripheral ostectomy.

## Introduction

In 2005, the World Health Organization (WHO) changed the term “parakeratinized odontogenic keratocyst” to “keratocystic odontogenic tumor” (KCOT) (1). However, WHO consensus group suggested that there was insufficient evidence to support a neoplastic origin of the KCOT and decided KCOT was removed from the odontogenic tumor classification and odontogenic keratocyst remains the most appropriate name for this lesion in the new 4th edition (2). OKC is a benign intraosseous lesion with invasive and aggressive behavior. It comprises approximately 2–21.8% of all jaw cysts (3-5). It is associated with a genetic mutation that may be also associated with the nevoid basal cell carcinoma syndrome (NBCCS), which is characterized by multiple OKCs in the jaws.

Because of high recurrence rates, ranging from 0% to 62% (6-8), there are different treatment options for OKCs. These treatment modalities have been broadly divided into two main categories: conservative approaches, including simple enucleation with or without curettage, decompression, or marsupialization, and aggressive approaches, including enucleation with peripheral ostectomy, enucleation with Carnoy’s solution, cryotherapy, and resection (en-bloc or marginal) (5,9).

Although OKCs are invasive and aggressive lesions, researchers are still seeking the best treatment option that would result in minimal morbidity because of the benign nature of the disease. Resection has the lowest recurrence rate among the various treatment options for OKCs; however, compared with other treatment modalities, it is associated with morbidities such as facial asymmetry and the loss of jaw continuity. Therefore, resection is suggested for large and recurrent lesions in difficult anatomic locations (10).

The purpose of the present study was to analyze the recurrence rate of OKCs treated by enucleation with peripheral ostectomy in the period between 2001 and 2015 at a single institution. Factors associated with recurrence will be discussed, and the clinical outcomes of peripheral ostectomy will be compared to those of other treatment options.

## Material and Methods

This retrospective study was performed at Hacettepe University, Faculty of Dentistry, Ankara, Turkey. An electronic search of the database of the Hacettepe University, Faculty of Medicine, Department of Pathology, to analyze the recurrence rate of OKCs treated by enucleation with peripheral ostectomy in the period between 2001 and 2015 was undertaken and included the following terms: “odontogenic keratocyst” or “keratocystic odontogenic tumor” or “odontogenic cyst”. In total, 600 patients with a tentative diagnosis of OKC were studied, and a definitive diagnosis for all patients was made according to the histopathological records. Of these, 89 patients were confirmed to have a histological diagnosis of odontogenic keratocysts. All histological slides were reevaluated according to the WHO criteria. Demographic, clinical, radiographic, and histologic data were collected for each patient.

In this study, localization of the OKC was divided in five categories: 1) anterior maxilla, including the region between the canines; 2) posterior maxilla, including the region from the first premolar to maxillary tuberosity; 3) anterior mandible, including the region between the canines; 4) posterior mandible, including the region from the first premolar to the third molar; and 5) mandibular ramus, from the angulus mandible to the sigmoid notch. The patients were recalled to record any recurrence based on clinical and radiographical examinations. Of the 89 patients, 38 were reached by phone and 51 patients could not be contacted because of a lack of phone records and contact addresses. Among these 38 patients, 3 were excluded because of an established diagnosis of NBCCS because of the synchronous presence of multiple OKCs, 3 were excluded because they died in the interim, and 5 were excluded because their definitive diagnosis was an orthokeratinized odontogenic cyst. Altogether, 27 patients diagnosed as OKC were re-examined to determine the recurrence after enucleation with peripheral ostectomy. All patients were examined clinically and underwent panoramic radiography. If necessary, cone-beam computed tomography images were taken.

Data were analyzed using the SPSS package to calculate mean and median values, percentages, and correlations. Different variables were compared using the chi-square test. *p* ≤ 0.05 was considered statistically significant.

## Results

The diagnosis of odontogenic keratocyst accounted for 14.8% of all jaw cysts in this search. A total of 81 patients (33 female and 48 male) with OKCs were evaluated in this study; 3 patients with NBCCS and 5 with orthokeratinized odontogenic keratocysts were excluded from the demographic data. The mean age at the time of diagnosis was 42 years, ranging from 10 to 83 years. The male:female ratio was 1:0.7. For female patients, the mean age was 39 years (range, 14-74 years), and for male patients, the mean age was 44.5 years (range, 10-83 years) ( Table 1).

**Table 1:**
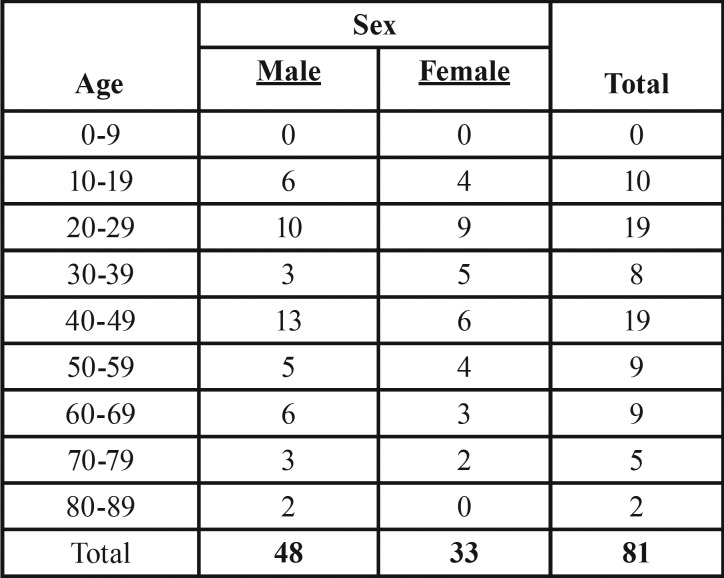
Distribution of odontogenic keratocyst patients.

**Figure 1:**
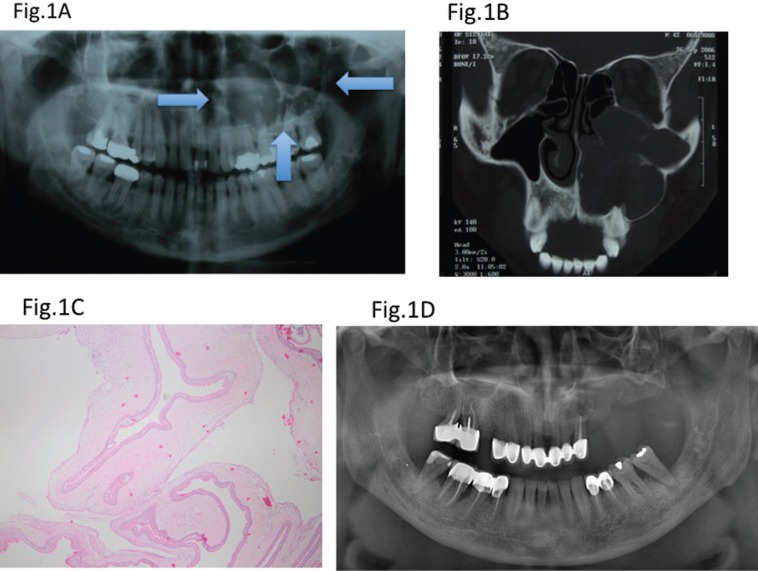
A) Multilocular radiolucent lesion in the posterior left maxillary region; B) Computed tomography (CT) images revealed that lesion had expanded the floor of the orbita and collapsed the left nasal passage; C) Thin, parakeratotic epithelial lining covering the cyst cavity was observed. The fibrous wall is devoid of inflammatory cells, and a small daughter cyst is seen within the fibrous wall. D) A panoramic radiography control after 8 years.

All patients were treated by enucleation and peripheral ostectomy. The median follow-up period of the patients was 5 years (range, 1-12 years). In 48.1% of the patients, the teeth associated with the lesion were extracted; in 37% of them, the teeth were endodontically treated before the surgery and apical resection was performed at the time of the surgery; and in 14.8% of them, the teeth were left alone. Figure 1 shows a case of a OKC in which the teeth associated with the lesion were extracted.

-Follow-up data

The recurrence rate, lesion location, teeth associated with the lesion, and follow-up period are reported for 27 patients. The posterior mandibular region was the most common location (41%), followed by mandibular ramus and maxillary posterior region ( Table 2).

**Table 2:**
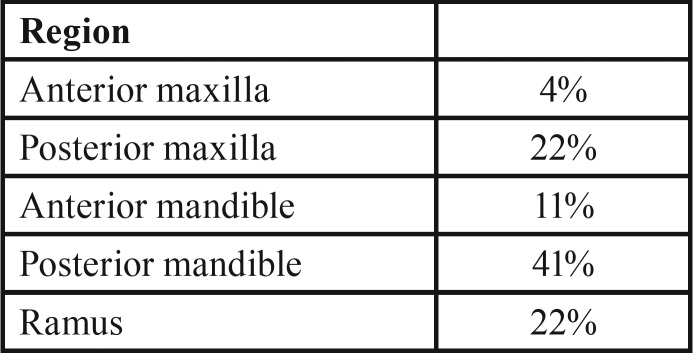
Location of odontogenic keratocyst %.

The recurrence rate was 14.8% (4 patients). All recurrent OKCs were observed in the mandible: 2 in the anterior mandible and 2 in the posterior region. The relationship between location of recurrence and recurrence rate was not statistically significant (*p* = 0.559).

**Figure 2:**
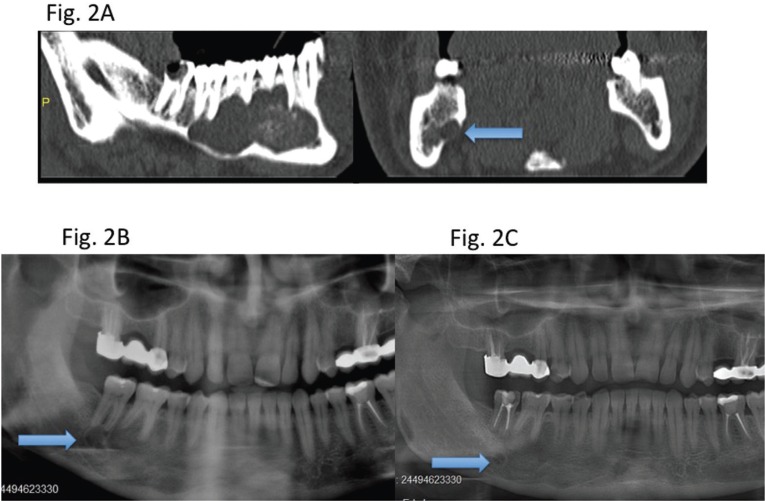
Recurrence due to a satellite cyst in the follow-up period. A) CT images showing the original lesion. The blue arrow shows a part of the cyst located lingually. B) First recurrence on the left second molar 5 years later. C) A satellite cyst was observed on the panoramic radiograph 2 years later after enucleation with peripheral ostectomy was performed for the recurrent case.

**Figure 3:**
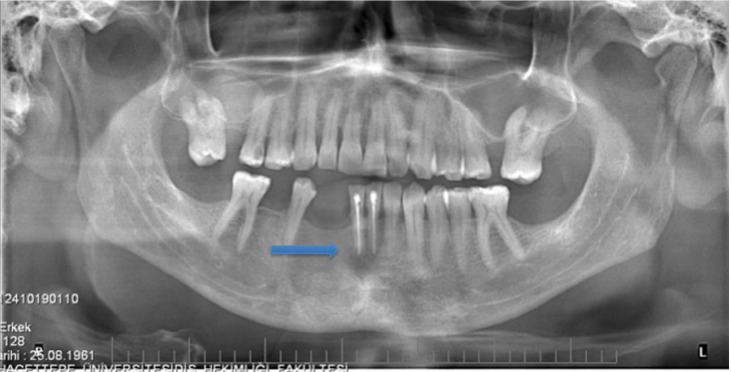
A patient with a recurrence surrounding the mandibular incisor teeth treated endodontically before the operation, after four years.

In recurrent cases, 1 tooth involved in the lesion was extracted, 2 teeth were treated endodontically, and 1 tooth was left alone. There was no statistically significant difference between the recurrence rate and the treatment protocol of the teeth (*p* = 0.579). During the follow-up period, the case in which the tooth was left alone was determined to have had a satellite cyst (Fig. 2). In another recurrent case, the lesion reappeared in close proximity to the teeth that were endodontically treated before the surgery (Fig. 3).

## Discussion

This retrospective study evaluated the recurrence rate of OKC in patients treated with enucleation and peripheral ostectomy between 2001 and 2015. The mean age of the patients was 42 years and male:female ratio was 1:0.7 at the time of diagnosis. The posterior mandibular area was the most common site. These clinical characteristics correlated with those reported in the literature (3,11,12).

Traditional enucleation is reported to be the most common surgical option for the treatment of OKCs in the literature but it shows higher recurrence rates because of the difficulties associated with removing the lesion in one piece (11). These epithelial residues result in the development of satellite cysts. The presence of a daughter cyst (from outpouchings of the main cyst lining) also increases the risk of recurrence (3,11). Therefore, enucleation with various adjuvant therapies has been tried to prevent recurrence. However, the best treatment modality remains controversial because of the lack of randomized controlled trials to prove the lowest recurrence rate with minimal morbidity.

Enucleation with peripheral ostectomy refers to surgical removal of the lesion by enucleation, followed by a reduction in peripheral bone with a powered hand-piece to remove the entire lesion without leaving any macroscopic remnants (13). Although enucleation with peripheral ostectomy is classified as an aggressive treatment approach, it has minimal morbidity compared with resection and enucleation with Carnoy’s solution. All patients in the present study were treated by enucleation with peripheral ostectomy. After enucleation of the lesion, a surgical hand-piece with a large round drill was used to remove the peripheral bone in the cavity under copious irrigation. At follow-up, all patients were satisfied and did not report complication, such as permanent paresthesia or functional and cosmetic defects.

In several systematic reviews, the recurrence rate of OKCs after enucleation with peripheral ostectomy has been reported to range from 17.4% to 18.2% (5,9,11,13). This is significantly higher than the recurrence after resection, ranging from 1.85% to 2.2%, and recurrence upon application of Carnoy’s solution, ranging from 4.8% to 5.3% (10,14). Despite the low recurrence rate associated with radical resection of OKCs, the surgical outcomes lead many clinicians to consider this treatment as too aggressive. Carnoy’s solution, the application of which is an alternative treatment option, is a cauterizing agent containing ethanol, chloroform, glacial acetic acid, and ferric chloride (15). Exposure to chloroform has been associated with cancer and reproductive toxicity (16). Therefore, in the United States, the Food and Drug Administration has banned the use of chloroform in Carnoy’s solution and many surgeons used a modified version of Carnoy’s solution (without chloroform) for the treatment of OKCs. Dashow *et al.* evaluated the effect of modified Carnoy’s solution compared to that of the original Carnoy’s solution with respect to the recurrence rate of OKCs; they reported a 10% recurrence rate of OKCs with Carnoy’s solution versus 35% with modified Carnoy’s solution, which is a statistically significant difference (17).

In the present study, the recurrence rate was 14.8% in 27 patients treated with enucleation with peripheral ostectomy with a median 5-year follow-up period.

The mean time between the primary treatment and the recurrence was 4.5 years, ranging from 3 to 6 years in the present study. It is well known that OKCs can recur at any time but most present within the first 5–7 years (11). Therefore, the follow-up period should be prolonged to at least 10–15 years.

In 2 patients, the recurrences were observed in edentulous alveolar bone, and it was suggested that these recurrences were related to inadequate surgical treatment. In the third case, a part of the whole cyst was left in the lingual side, which was not noticed at the time of first surgery (Fig. 2). In the last case, recurrence was seen at the tooth apices in the anterior mandible on the panoramic radiograph (Fig. 3). In the present study, no statistically significant association was found between the recurrence rate and treatment method for teeth associated with the lesion (*p* = 0.579). However, the preservation of a tooth involved in the lesion has been reported to increase the risk of recurrence, and extraction of the affected tooth in cases of root involvement has been suggested (18). In the present study, the recurrent cases were again treated with enucleation with peripheral ostectomy, and the teeth involved in the lesion were extracted.

In this study, all recurrences were observed in the mandible, and no statistically significant association between the recurrence rate and tumor site was detected (*p* = 0.559). In the literature, the relation between tumor localization and rate of recurrence is also controversial, but most recurrences were reported to be in the mandibular molar area (13). The authors thought that this may be because the most common site for OKCs is the mandible.

One of the possible limitations of the present study is that the relationship among tumor size, radiographic characteristics of the lesion, and recurrence rate was not evaluated. MacDonald *et al.* compared recurrent and non-recurrent OKCs according to age, sex, tumor size, localization, and radiological appearance and they did not find and statistically significant association between the recurrent and non-recurrent OKCs according to the tumor size and radiological appearance of the lesion (19). This finding was confirmed by different clinical studies as well (11,18).

This retrospective study analyzed the recurrence rate of OKCs treated with enucleation with peripheral ostectomy. The authors in this study suggest that enucleation with peripheral ostectomy may safely treat OKCs with minimal morbidity. It is thought that the recurrence rate can be decreased with careful preoperative radiographic examination and meticulous surgery performed by experienced surgeons.
